# Supplementary material for: Overexpression of an Antioxidant Enzyme APX1 in cpr5 Mutant Restores its Pleiotropic Growth Phenotype
Source: Antioxidants (Basel). 2023 Jan 28;12(2):301. doi: 10.3390/antiox12020301 (PMC9952838; doi:10.3390/antiox12020301)
Supplement: Supplementary file 1 [file antioxidants-12-00301-s001.zip › antioxidants-2113921-supplementary.pdf]

Supplemental Figures

A

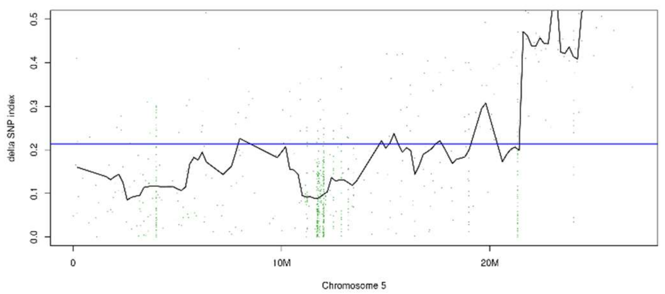

B

Gene ID	Position (bp)	Mutant category	SNP	Amino-acid change	Annotation
AT5G61320 *CYP89A3*	24656379	exonic	C260T	A87V	Polypeptide of cytochrome p450 family acting on paired donors, with incorporation or reduction of molecular oxygen
AT5G64930 *CPR5*	25946738	exonic	C842T	A281V	A component of the nuclear pore complex which participates in signal transduction pathways involved in plant growth and defense
AT5G65770 *CRWN4*	26315557	exonic	G2980A	A994T	Nuclear periphery-located protein with functions in protection from oxidative damage, control of plant growth and defense.

Figure S1. Molecular characterization of *DELT9*. (A) ΔSNP-index plot of *Chr5* generated by whole-genome sequencing. Green dots correspond to each ΔSNP-index. The black line represents the average ΔSNP-index at 1-Mb intervals with 100-kb increments. The candidate region is above the blue line indicating cutoff values. **(B)** List of candidates. Three nonsynonymous SNPs were identified in the candidate regions.

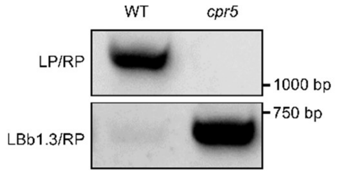

Figure S2. Genotyping of *cpr5* T-DNA insertion mutants. *cpr5* homozygotes were identified by PCR. The primers used for amplification are listed in Table S1.

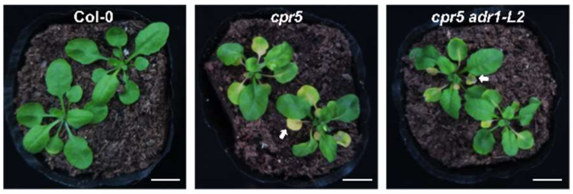

Figure S3. Mutation of ADR1-L2 does not suppress the premature leaf senescence in *cpr5*. Representative photographs of 4-wk-old plants. White arrows indicate early senescence leaves. Scale bars, 1 cm.

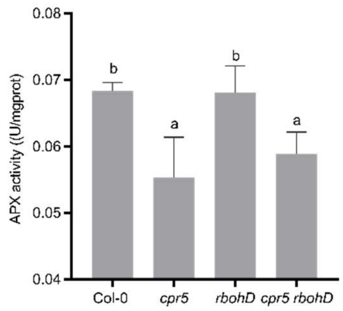

Figure S4. Introduction of *rbohD* into *cpr5* did not recover the reduced APX activity. APX activity was measured in the 5th–7th rosette leaves from 4-wk plants. Data are the mean ± SD (n = 4). Letters above bars indicate significantly different values among groups ($P < 0.05$; one-way ANOVA). The experiment was performed twice with similar results each time.

Table S1. Primers used in this study

Primer name	Sequence (5' to 3')	Function
Salk_074631 LP	TTGTTGCAGTGATAGCACCAG	Genotyping for cpr5 T-DNA homozygote
Salk_074631 RP	TTTGGGTGCAGATATTTTTCG	
LBb1.3	ATTTTGCCGATTTCGGAAC	T-DNA genotyping
CPR5-GW-F	GGGGACAAGTTTGTACAAAAAAGCAGGCTACATGGAAGCCCTCCTCCTCCCT	Cloning for 35S::CPR5-3×HA
CPR5-GW-R	GGGGACCACTTTGTACAAGAAAGCTGGGTCAGCATAGTCAGACCCACCAT	
WRKY25-qF	GGTTGTGGAGTGAAGAAGCA	qRT-PCR
WRKY25-qR	TCGTGATTGTGTCTTCCTTCA	
WRKY53-qF	TCACCGAGCGTACAACTTATTCC	
WRKY53-qR	CGTTTATCGATGCCGGAGATT	
WRKY75- qF	CCGTCAAGAACAACAAGTTCCC	
WRKY75- qR	TATGCTCGAAGTTTTCGGTGGA	
GSTF2- qF	CCAGCTTCCGAGAAGGTTCAGTG	
GSTF2- qR	GCCAAAGATACTCTCAAGAGCATACAC	
GSTF3- qF	CTTTGAGCTCGTTCATGTTGAA	
GSTF3- qR	TTCGTATCGGTGAGCTATGTAC	
GSTF7- qF	TGAAGATGGAGACTTCAAGCTT	
GSTF7- qR	CATGCGATTCAATTTCAATGCC	
AOX1a- qF	GTTTCAGGCCATGGGAAACG	
AOX1a- qR	CAAGAAACGTCGTTGGAACATGA	
AOX1d- qF	CCAAATGGTACGAACGAGCG	
AOX1d- qR	ACGATGAGCAAGTTTGGGTGA	
MDAR3- qF	CTGAAGCCTGGTGAACTCGC	
MDAR3- qR	GGTCGGATTGACTTCGAGGTC	
SAG13- qF	GGGCTTGGGAGAGAACTCAA	

SAG13- qR	TGGCTAGTTCCTCCACCAC	
